# Expression Changes and Impact of the Extracellular Matrix on Etoposide Resistant Human Retinoblastoma Cell Lines

**DOI:** 10.3390/ijms21124322

**Published:** 2020-06-17

**Authors:** Jacqueline Reinhard, Natalie Wagner, Miriam M. Krämer, Marvin Jarocki, Stephanie C. Joachim, H. Burkhard Dick, Andreas Faissner, Vinodh Kakkassery

**Affiliations:** 1 Department of Cell Morphology and Molecular Neurobiology, Faculty of Biology and Biotechnology, Ruhr-University Bochum, Universitaetsstrasse 150, 44780 Bochum, Germany; natalie.wagner@rub.de (N.W.); miriam.kraemer@rub.de (M.M.K.); marvin.jarocki@rub.de (M.J.); andreas.faissner@rub.de (A.F.); 2 Experimental Eye Research Institute, University Eye Hospital, Ruhr-University Bochum, In der Schornau 23-25, 44892 Bochum, Germany; stephanie.joachim@rub.de (S.C.J.); Burkhard.Dick@kk-bochum.de (H.B.D.); 3 Department of Ophthalmology, University of Luebeck, Ratzeburger Allee 160, 23538 Luebeck, Germany

**Keywords:** chemotherapy resistance, etoposide, extracellular matrix, integrins, retinoblastoma, WERI-RB1, Y79

## Abstract

Retinoblastoma (RB) represents the most common malignant childhood eye tumor worldwide. Several studies indicate that the extracellular matrix (ECM) plays a crucial role in tumor growth and metastasis. Moreover, recent studies indicate that the ECM composition might influence the development of resistance to chemotherapy drugs. The objective of this study was to evaluate possible expression differences in the ECM compartment of the parental human cell lines WERI-RB1 (retinoblastoma 1) and Y79 and their Etoposide resistant subclones via polymerase chain reaction (PCR). Western blot analyses were performed to analyze protein levels. To explore the influence of ECM molecules on RB cell proliferation, death, and cluster formation, WERI-RB1 and resistant WERI-ETOR cells were cultivated on Fibronectin, Laminin, Tenascin-C, and Collagen IV and analyzed via time-lapse video microscopy as well as immunocytochemistry. We revealed a significantly reduced mRNA expression of the proteoglycans *Brevican*, *Neurocan,* and *Versican* in resistant WERI-ETOR compared to sensitive WERI-RB1 cells. Also, for the glycoproteins *α1-Laminin*, *Fibronectin*, *Tenascin-C,* and *Tenascin-R* as well as *Collagen IV*, reduced expression levels were observed in WERI-ETOR. Furthermore, a downregulation was detected for the matrix metalloproteinases *MMP2*, *MMP7*, *MMP9*, the tissue-inhibitor of metalloproteinase *TIMP2*, the Integrin receptor subunits *ITGA4*, *ITGA5* and *ITGB1,* and all *receptor protein tyrosine phosphatase β/ζ* isoforms. Downregulation of *Brevican*, *Collagen IV*, *Tenascin-R*, *MMP2*, *TIMP2,* and *ITGA5* was also verified in Etoposide resistant Y79 cells compared to sensitive ones. Protein levels of Tenascin-C and MMP-2 were comparable in both WERI cell lines. Interestingly, Fibronectin displayed an apoptosis-inducing effect on WERI-RB1 cells, whereas an anti-apoptotic influence was observed for Tenascin-C. Conversely, proliferation of WERI-ETOR cells was enhanced on Tenascin-C, while an anti-proliferative effect was observed on Fibronectin. In WERI-ETOR, cluster formation was decreased on the substrates Collagen IV, Fibronectin, and Tenascin-C. Collectively, we noted a different ECM mRNA expression and behavior of Etoposide resistant compared to sensitive RB cells. These findings may indicate a key role of ECM components in chemotherapy resistance formation of RB.

## 1. Introduction

Retinoblastoma (RB) has an incidence rate of 1 in 15,000–20,000 live births. With approximately 8,000–9,000 new cases every year, it is the most common malignant pediatric ocular tumor worldwide [1,2,3,4]. RB is highly curable when diagnosed early and treated appropriately. Due to an improvement of therapeutic strategies over recent years, the survival rate is nearby 99% in developing countries, while in Africa or Asia death rates are still high [5,6,7].

Two types of RB can be distinguished, namely the spontaneous and the inherited form. Both forms are caused by an inactivation of the retinoblastoma 1 (RB1) tumor suppressor gene. Therefore, RB displays an excellent model for RB1 protein eliminated tumors.

RB tumor usually manifests in children before the age of five. All RB treatment options follow the priority to preserve the child’s life. The highest chance to reduce lethality dramatically from 95% to 5% in RB, is to surgically remove the affected eye [8]. To prevent eye removal in children, further chemotherapy, cryotherapy, photocoagulation, radiotherapy, or combined strategies were introduced over the last decades [8,9]. Especially chemotherapy is effective in tumor control. Low rates of secondary CNS malignancies in RB are a major benefit of chemotherapy in comparison to radiation therapy [10,11,12,13,14,15,16,17]. Therefore, chemotherapy has been established as first-line therapy in RB. Nevertheless, chemotherapy resistance, also for Etoposide, limits eye preserving therapy [18].

Several studies described that a dysregulation of the extracellular matrix (ECM) plays a crucial role in tumor growth, progression, metastasis, and angiogenesis [19,20,21,22,23,24]. Also, ECM interacting enzymes such as matrix metalloproteinases (MMPs) and tissue-inhibitors of metalloproteinases (TIMPs) as well as receptors like Integrins represent important modifying components in tumor tissue [25,26]. Moreover, studies indicate that the ECM composition might influence resistance development to various chemotherapeutic drugs [27,28]. Thus, understanding the exact ECM composition in RB and other tumors and its role in chemotherapeutic drug resistance is of great interest to improve and develop novel treatment strategies.

Due to the cancer stem cell (CSC) hypothesis, it was proposed that cancer arises from a small population of self-sustaining cancer cells, which exhibit self-renewal and maintenance capacity. Regarding this finding, we recently reviewed that CSCs and neural stem cells share a similar ECM compartment [29]. Preceding studies also reported the presence of stem cells in human RB [30,31,32,33]. Moreover, Jia et al. noted that multidrug resistance might play a key role in RB CSCs isolated from the WERI-RB1 cell line [18]. Expression of multidrug resistance proteins was also described in human primary RB Y79 cultures after exposure to chemotherapeutic agents, including Etoposide [34]. In this regard, an improved knowledge of chemotherapy resistance is crucial when conceptualizing new treatment strategies.

The aim of the present study was to evaluate the potential role of the ECM in retinoblastoma chemotherapy resistance. Accordingly, we analyzed different human RB cell lines. The parental cell line WERI-RB1 was initially established by McFall and colleagues and is sensitive to Etoposide [35]. The WERI-ETOR cell line represents a described Etoposide resistant subclone of this parental cell line [36,37,38]. Additionally, the parental Y79 RB cell line, first described by Reid et al. [39], and a resistant Y79 subclone were investigated.

Via PCR, expression levels of various ECM proteoglycans and glycoproteins were analyzed and compared in the Etoposide sensitive and resistant RB cell lines. Furthermore, the mRNA regulation of ECM degrading MMPs and counteracting TIMPs as well as of important ECM receptors, namely Integrin receptor subunits and receptor protein tyrosine phosphatase (RPTP) β/ζ isoforms was explored. Additionally, protein levels of Tenascin-C and MMP-2 were examined via Western blot analyses in WERI cell lines. Finally, the impact of various ECM components on cellular death, proliferation and cluster formation was investigated by time-lapse video microscopy as well as immunocytochemistry.

## 2. Results

### 2.1. CSPG mRNA Expression in WERI-RB1 and WERI-ETOR

**Figure 1:**
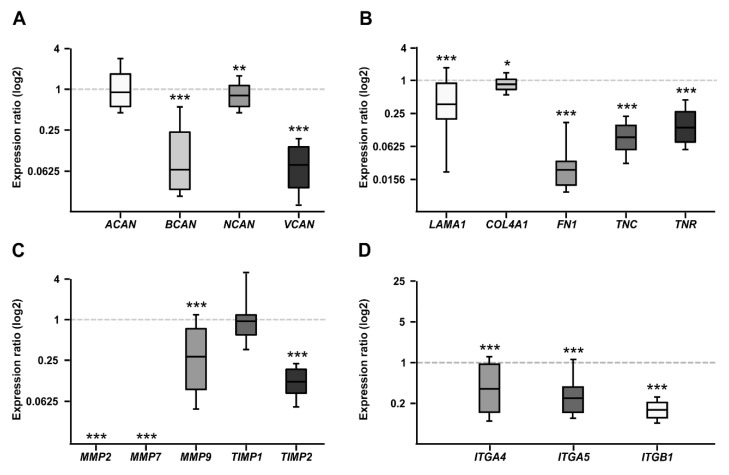
RT-qPCR analyses of relative CSPG, extracellular matrix (ECM) glycoprotein, matrix metalloproteinases (MMPs), tissue-inhibitor of metalloproteinases (*TIMPs),* and Integrin mRNA expression in the WERI-ETOR compared to the WERI-RB1 cell line. (**A**) In the resistant WERI-ETOR cell line, significantly reduced levels of *Brevican* (*BCAN*), *Neurocan* (*NCAN*), and *Versican* (*VCAN*) were observed. In contrast, *Aggrecan* (*ACAN*) expression was comparable in both WERI cell lines. (**B**) A significant downregulation was observed for all ECM glycoproteins (*α1-Laminin* (*LAMA1*), *Fibronectin* (*FN1*), *Tenascin-C* (*TNC*), and *Tenascin-R* (*TNR*)) as well as for *Collagen IV* (*COL4A1*). (**C**) Significant lower expression levels were detected for *MMP2*, *MMP7*, *MMP9,* and *TIMP2*. While *TIMP1* expression was similar in both WERI cell lines. (**D**) In the WERI-ETOR cell line, significantly reduced levels of integrin receptor subunits *ITGA4, ITGA5,* and *ITGB1* were noted. Values are median ± quartile + maximum/minimum. The dotted line in the graphs represents the relative expression level of the WERI-RB1 cell line. * *p* < 0.05; ** *p* < 0.01; *** *p* < 0.001; *n* = 10/group.

Proteoglycans are major constituents of the ECM. In order to characterize the expression of the CSPGs *Aggrecan* (*ACAN*), *Brevican* (*BCAN*), *Neurocan* (*NCAN*), and *Versican* (*VCAN*) in Etoposide sensitive and resistant cells, quantitative real-time PCR (RT-qPCR) analyses were performed (Figure 1A). The analyses revealed a comparable mRNA expression level for *ACAN* in both cell lines (0.758-fold; *p* = 0.16). In contrast, a prominent downregulation of the *BCAN* (0.064-fold; *p* < 0.001) as well as *VCAN* (0.075-fold; *p* < 0.001) mRNA expression level was observed in the resistant WERI-ETOR compared to the sensitive WERI-RB1 cells. Also, for *NCAN*, a significantly reduced expression level was found in the WERI-ETOR cell line (0.682-fold; *p* = 0.003).

### 2.2. Expression of ECM Glycoproteins in WERI-RB1 and WERI-ETOR

Next, the mRNA expression of the glycoproteins *α1-Laminin* (*LAMA1*), *Fibronectin* (*FN1*), *Tenascin-C* (*TNC*), and *Tenascin-R* (*TNR*) was analyzed in both WERI cell lines (Figure 1B). Additionally, the expression of *Collagen IV* (*COL4A1*) was investigated. As revealed by RT-qPCR, both *LAMA1* (0.373-fold; *p* = 0.001) and *FN1* (0.023-fold; *p* < 0.001) displayed a significantly lower expression in WERI-ETOR compared to WERI-RB1 cells. Also, for *COL4A1* a reduced mRNA expression level was detected in the WERI-ETOR cell line (0.852; *p* = 0.046). For both analyzed Tenascins, namely *TNC* (0.091-fold; *p* = 0.001) and *TNR* (0.137-fold; *p* < 0.001), the mRNA expression level was significantly lower in WERI-ETOR cells.

**Figure A1:**
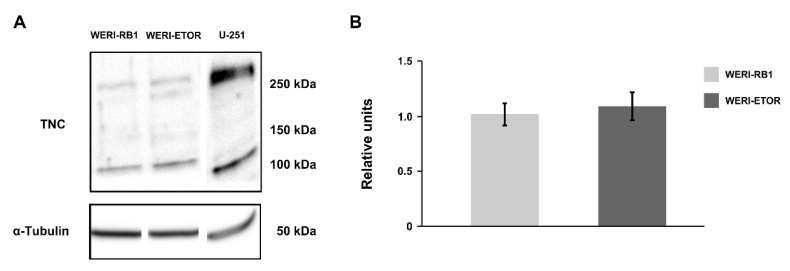
Western blot analyses of TNC in the WERI-RB1 and WERI-ETOR cell line. (**A**) Representative Western blot analysis using protein lysates of WERI-RB1 and WERI-ETOR cells. Lysate of the glioblastoma cell line U-251 was used as positive control. Distinct bands at > 250, > 150, and > 100 kDa were detectable for TNC. α-Tubulin was detectable at ~50 kDa. (**B**) A comparable TNC protein level was noted in both WERI cell lines. Data are shown as mean ± SEM. *n* = 4/group.

To further investigate TNC protein levels, Western blot analyses were performed. However, similar TNC protein levels (WERI-RB1: 1.01 ± 0.51 relative units; WERI-ETOR: 1.09 ± 0.63 rel. units; *p* = 0.84) were found in both WERI cell lines (Figure A1).

### 2.3. Expression of MMPs and TIMPs in WERI-RB1 and WERI-ETOR

Remodeling of the ECM is primarily mediated by MMPs and counteracting TIMPs. MMPs, and TIMPs play a key role in tumor cell adhesion [40]. Therefore, RT-qPCR analyses were performed to analyze the mRNA expression pattern of *MMP-2* (*MMP2*), *MMP-7* (*MMP7*), and *MMP-9* (*MMP9*) as well as *TIMP-1* (*TIMP1*) and *TIMP-2* (*TIMP2*; Figure 1C). Interestingly, *MMP2* and *MMP7* mRNA expression was detectable at lowest levels in WERI-ETOR cells (*p* < 0.001). Also, the expression of *MMP9* was significantly decreased in the WERI-ETOR compared to the WERI-RB1 cell line (0.314-fold; *p* < 0.001). The expression of *TIMP1* was comparable in both WERI groups (1.038-fold; *p* = 0.09). In contrast, *TIMP2* expression was significantly reduced in WERI-ETOR cells (0.135-fold; *p* < 0.001).

**Figure A2:**
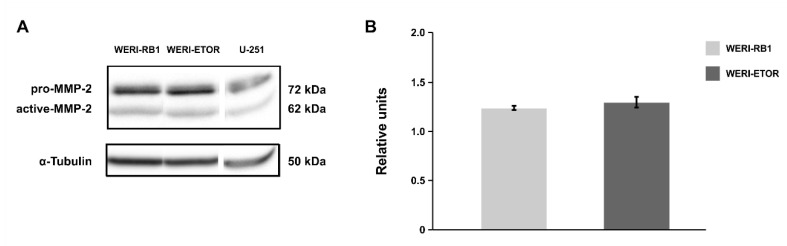
Western blot analyses of the MMP-2 protein in the WERI-RB1 and WERI-ETOR cell line. (**A**) Representative Western blot analysis using protein lysates of WERI-RB1 and WERI-ETOR cells. Lysate of the glioblastoma cell line U-251 was used as positive control. Pro- and active-MMP-2 protein was detectable at 72 and 62 kDa, respectively. α-Tubulin was detectable at ~50 kDa. (**B**) A comparable MMP-2 protein concentration was measured in both WERI cell lines. Data are shown as mean ± SEM. *n* = 4/group.

In order to investigate MMP-2 protein levels, Western blot analyses were conducted. Here, pro- and active-MMP-2 proteins were observed in both cell lines at a comparable level (WERI-RB1: 1.23 ± 0.03 rel. units; WERI-ETOR: 1.29 ± 0.06 rel. units; *p* = 0.63; Figure A2).

### 2.4. Expression of Integrin Receptor Subunits in WERI-RB1 and WERI-ETOR

Integrins represent important ECM receptors and have been implicated in tumor progression as well as tumor cell migration and proliferation [41,42]. To better understand the potential role of Integrins in RB and resistance development, the mRNA expression levels of the Integrin receptor subunits α4 (*ITGA4*), α5 (*ITGA5*), and *β1* (*ITGB1*) were analyzed in both WERI cell lines via RT-qPCR (Figure 1D). Our examination of *Integrin* levels revealed a significantly reduced mRNA expression of α*4-Integrin* (*ITGA4*; 0.285-fold; *p* = 0.03), α*5-Integrin* (*ITGA5;* 0.198-fold; *p* < 0.001) and *β1-Integrin* (*ITGB1;* 0.126-fold; *p* < 0.001) in WERI-ETOR cells.

### 2.5. Expression of CSPGs, ECM Glycoproteins, MMPs, TIMPs, and Integrin Receptor Subunits in Etoposide Sensitive and Resistant Y79 RB cells

**Figure A3:**
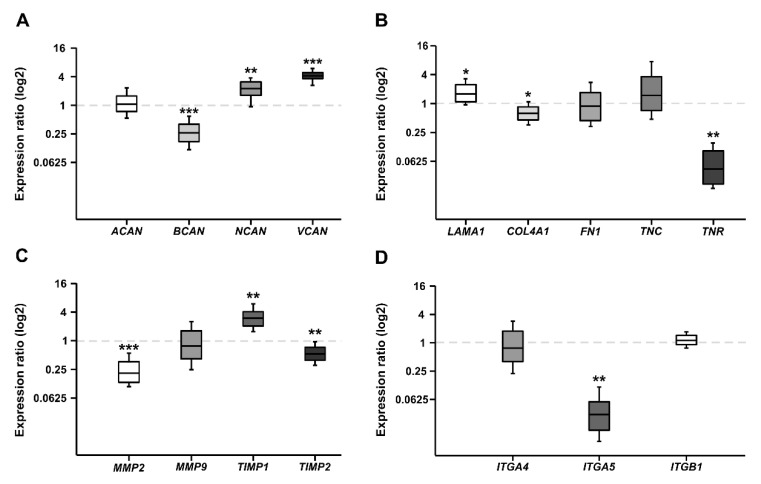
RT-qPCR analyses of relative CSPG, ECM glycoprotein, MMP, TIMP, and Integrin mRNA expression in Etoposide sensitive and resistant Y79 cell lines. (**A**) A similar *ACAN* expression was observed in both Y79 cell lines. Significantly reduced *BCAN* levels were found in resistant compared to sensitive Y79 cells. In contrast, *NCAN* and *VCAN* expression levels were significantly higher in the resistant Y79 cell line. (**B**) The expression of *LAMA1* was significantly upregulated in the resistant Y79 cell line. While *COL4A1* and *TNR* were significantly downregulated in resistant Y79 cells. A comparable *FN1* and TNC mRNA level was observed in both Y79 cell lines. (**C**) Significantly reduced expression levels were found for *MMP2* and *TIMP2*. While *MMP9* mRNA expression was comparable in both cell lines. *TIMP1* was significantly upregulated in the resistant cell line. (**D**) The resistant Y79 cells showed a significantly reduced *ITGA5* expression. *ITGA4* and *ITGB1* expression was similar in both groups. Values are median ± quartile + maximum/minimum. The dotted line in the graphs represents the relative expression level of the Etoposide sensitive Y79 cell line. * *p* < 0.05; ** *p* < 0.01; *** *p* < 0.001; *n* = 6/group.

In order to further explore the mRNA expression levels of CSPGs, ECM glycoproteins, MMPs, TIMPs and Integrin receptor subunits in an independent human RB cell line, we analyzed Etoposid sensitive and resistant Y79 cells by RT-qPCR (Figure A3).

As shown for the WERI-ETOR cell line, our analyses verified a significantly reduced expression level of the proteoglycan *BCAN* (0.262-fold; *p* < 0.001), *COL4A1* (0.625-fold; *p* = 0.018), and the ECM glycoprotein *TNR* (0.043; *p* = 0.001) in Etoposid resistant Y79 cells compared to the sensitive Y79 cell line. Also, *MMP2* (0.210-fold; *p* < 0.001), *TIMP2* (0.527-fold; *p* = 0.002) as well as *ITGA5* (0.029-fold; *p* = 0.003) displayed a reduced expression in this group. Furthermore, a comparable expression level for *ACAN* (1.062-fold; *p* = 0.761) was found in both Y79 cell lines, as noted for both WERI cell lines. Comparable to the effects in resistant WERI-ETOR cells, resistant Y79 cells often showed a slightly lower expression. However, overall a comparable *FN1* (0.888-fold; *p* = 0.686), *TNC* (1.463-fold; *p* = 0.353), *MMP9* (0.788-fold; *p* = 0.397), *ITGA4* (0.762-fold; *p* = 0.419), and *ITGB1* (1.130-fold; *p* = 0.254) expression level was seen. Contrary to the findings in the resistant WERI-ETOR cell line, expression of *NCAN* (2.207-fold; *p* = 0.005), *VCAN* (4.121-fold; *p* < 0.001), *LAMA1* (1.598-fold; *p* = 0.01) as well as of *TIMP1* (3.025-fold; *p* = 0.001) was significantly upregulated in the Y79 cell line. Although *MMP7* was expressed at lowest levels in the WERI-ETOR compared to the WERI-RB1 cells, its expression was not detectable in both Y79 cell lines.

### 2.6. Expression of RPTPβ/ζ Isoforms in WERI-RB1 and WERI-ETOR

**Figure 2:**
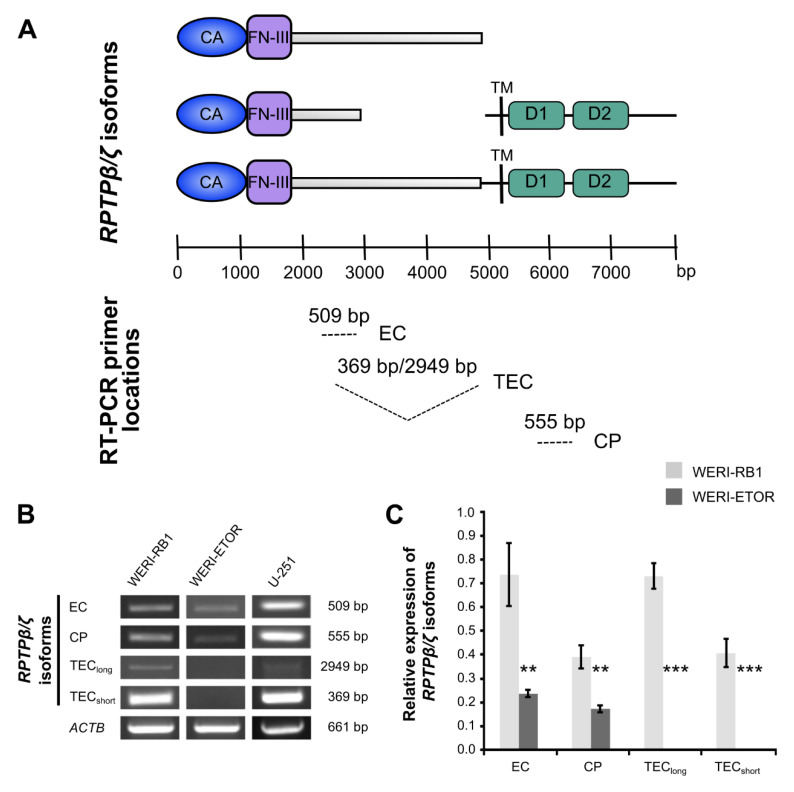
Semi-quantitative RT-PCR analyses of *RPTPβ/ζ* isoforms in WERI-RB1 compared to WERI-ETOR cells using various primer sets. (**A**) Scheme displays binding sites of specific *RPTPβ/ζ* (*PTPRZ1*) primer pairs for isoform amplification. All three of the RPTPβ/ζ isoforms, which were generated by alternative splicing, exhibit an extracellular carbonic anhydrase-like (CA) domain and Fibronectin-type-III (FN-III) repeats. A set of three distinct primers pairs (EC, TEC, and CP) marks the corresponding nucleotide sequence to demonstrate the resulting amplicon (modified according to Norman and colleagues [45]). (**B**) Using a set of three different primer pairs, the expression of *RPTPβ/ζ* isoforms was evaluated in both cell lines. The glioblastoma multiforme cell line U-251 served as positive control. The primer pair termed EC amplified a 509 bp product. This amplified product is part of all *RPTPβ/ζ* isoforms. A second primer pair, termed CP, amplified a 555 bp product, which corresponds to both *RPTPβ/ζ* receptor isoforms. Using this primer pair, expression of *RPTPβ/ζ_long_*_/*short*_ was verified in WERI-RB1 cells. In contrast, only little, if any expression, was observed in WERI-ETOR cells. A third primer pair, termed TEC, amplified a 369 bp (*RPTPβ/ζ_short_*) as well as a 2949 bp (*RPTPβ/ζ_long_*) fragment. Both transcripts were verified in the WERI-RB1 line but were absent in the WERI-ETOR cell line. (**C**) Densitometric measurements of band intensities followed by semi-quantitative analyses and relative quantification (normalized to *ACTB* expression) revealed a significant downregulation of all *RPTPβ/ζ* isoforms in WERI-ETOR compared to WERI-RB1 cells. Values are shown as mean ± SD. ** *p* < 0.01; *** *p* < 0.001; *n* = 5/group.

RPTPβ/ζ is another member of the CSPG family. By alternative splicing three different *RPTPβ/ζ* (*PTPRZ1*) isoforms are generated, namely the secreted splice variant Phosphacan and both receptor isoforms *RPTPβ/ζ*_long_ and *RPTPβ/ζ*_short_. Phosphacan/RPTPβ/ζ is an important binding partner for Tenascin-C [43,44]. To explore *RPTPβ/ζ* isoform mRNA expression in WERI-RB1 and WERI-ETOR cells, a set of three different primer pairs was used for semi-quantitative RT-PCR analyses (Figure 2A–C).

According to previous descriptions and as shown for each set schematically, these primer pairs bind to specific regions of the three *RPTPβ/ζ* isoforms (Figure 2A; [45]). The glioblastoma multiforme cell line U-251 served as positive control, since all *RPTPβ/ζ* isoforms are prominently expressed in different grades of glioma types [45].

A primer pair, termed EC, amplified a 509 bp product, which corresponds to a product present in all *RPTPβ/ζ* isoforms. By using this primer pair, semi-quantitative analyses revealed a significant downregulation in WERI-ETOR compared to WERI-RB1 (*p* = 0.002; Figure 2B,C). A second primer pair, termed CP, amplified a 555 bp fragment, located in the cytoplasmic part of *RPTPβ/ζ* and allows the identification of both *RPTPβ/ζ* receptor variants. Using this primer pair, expression of *RPTPβ/ζ_long_*/*RPTPβ/ζ_short_* was verified in WERI-RB1 cells. In contrast, only little, if any, expression, was observed in WERI-ETOR cells (*p* = 0.005; Figure 2B,C). TEC, a third primer pair, amplified a 2949 bp (*RPTPβ/ζ_long_*) and a 369 bp (*RPTPβ/ζ_short_*) fragment. Both transcripts were verified in the WERI-RB1 line, but, were not detected in the WERI-ETOR cell line (*p* < 0.001; Figure 2B,C). In sum, based on the RT-PCR analyses, *RPTPβ/ζ* isoforms were significantly downregulated in the WERI-ETOR cell line.

### 2.7. Impact of the ECM on WERI-RB1 and WERI-ETOR Cell Survival

**Figure 3:**
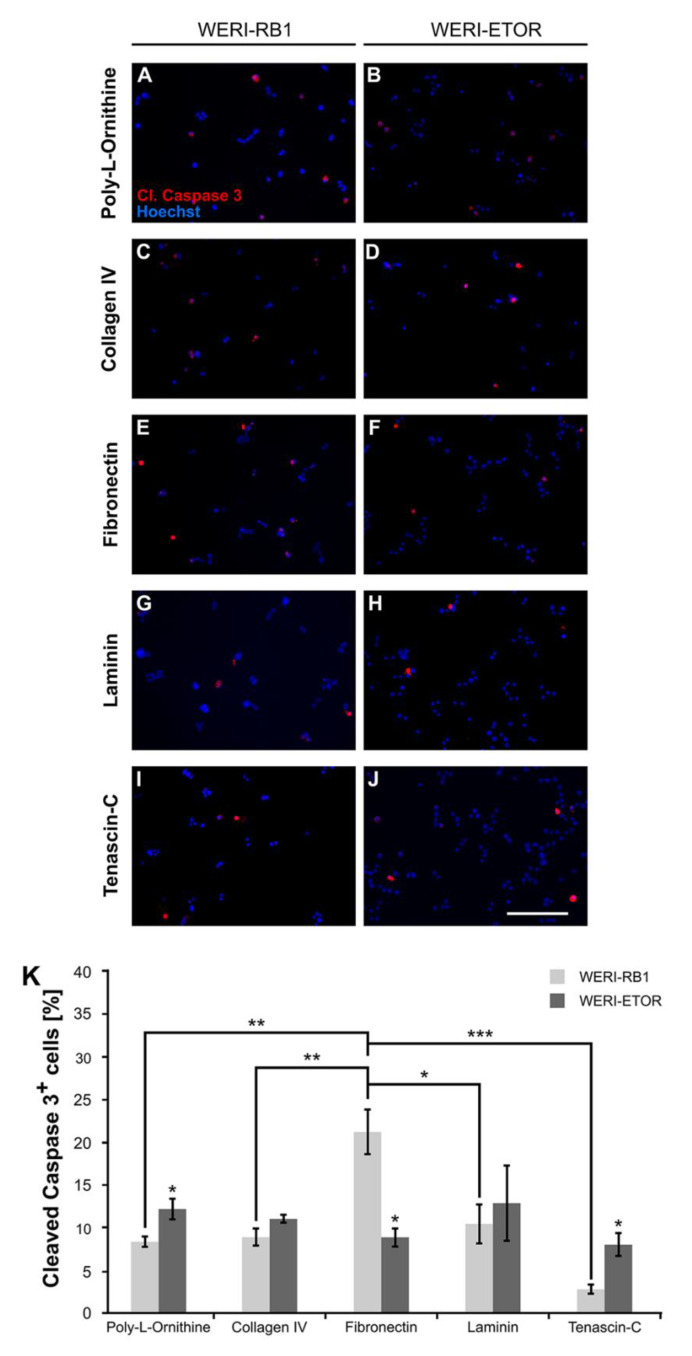
Immunocytochemical detection of apoptotic WERI-RB1 and WERI-ETOR cells cultivated on various ECM substrates. (**A–J**) Apoptotic cells were identified by cleaved Caspase 3 (cl. Caspase 3) immunostaining (red) and nuclear Hoechst co-staining (blue) in WERI-RB1 (**A,C,E,G,I**) and WERI-ETOR cells (**B,D,F,H,J**). (**K**) Significantly more cleaved Caspase 3^+^ cells were identified for the WERI-ETOR compared to the WERI-RB1 cell line when cultivated on the non-ECM control Poly-L-Ornithine or the ECM glycoprotein Tenascin-C. On Collagen IV and Laminin, the percentage of apoptotic WERI-RB1 and WERI-ETOR cells was comparable. In contrast, on Fibronectin, a significantly reduced percentage of cleaved Caspase 3^+^ cells was observed for WERI-ETOR compared to WERI-RB1 cells. Furthermore, our analyses of the WERI-RB1 cell line revealed a significant increased number of apoptotic cells on Fibronectin when compared to the other substrates. Data are shown as mean ± SEM. * *p* < 0.05; ** *p* < 0.01; *** *p* < 0.001; *n* = 3/group. Scale bar = 100 µm.

Next, the influence of various ECM substrates on apoptosis of WERI-RB1 and WERI-ETOR was investigated by immunocytochemical stainings after 2 days *in vitro* (div). Dissociated cells were cultivated on wells coated with either Poly-L-Ornithine (non-ECM control substrate) or Poly-L-Ornithine and Collagen IV, Fibronectin, Laminin, and Tenascin-C, respectively. Apoptotic cells were identified by cleaved Caspase 3 immunostaining and nuclear Hoechst co-staining, which allows to monitor nuclear condensation, cell shrinkage, and DNA fragmentation (Figure 3A–K).

On Poly-L-Ornithine, significantly more cleaved Caspase 3^+^ cells were identified for the WERI-ETOR (12.18 ± 1.23%) compared to the WERI-RB1 cell line (8.35 ± 0.61%; *p* = 0.048). Cultured on Collagen IV, the percentage of apoptotic WERI-RB1 and WERI-ETOR cells was about the same (WERI-RB1: 8.88 ± 0.98%; WERI-ETOR: 11.02 ± 0.44%; *p* = 0.13). However, on Fibronectin, a significantly decreased percentage of cleaved Caspase 3^+^ cells was observed for the WERI-ETOR (8.87 ± 1.08%) compared to the WERI-RB1 cell line (21.20 ± 2.64%; *p* = 0.012). For Laminin coating, the number of cleaved Caspase 3^+^ cells was similar in both cell lines (WERI-RB1: 10.44 ± 2.28%; WERI-ETOR: 12.87 ± 4.41%; *p* = 0.65). Interestingly, significantly more apoptotic WERI-ETOR cells (8.33 ± 1.33%) were identified on Tenascin-C compared to apoptotic WERI-RB1 cells (2.78 ± 0.53%; *p* = 0.02). Furthermore, our analyses of the WERI-RB1 cell line revealed a significantly increased number of apoptotic cells on Fibronectin when compared to Poly-L-Ornithine (*p* = 0.004), Collagen IV (*p* = 0.007), Laminin (*p* = 0.02), and Tenascin-C (*p* < 0.001). The WERI-ETOR cell line had a comparable apoptotic rate on all substrates (*p* > 0.05).

### 2.8. Influence of the ECM on WERI-RB1 and WERI-ETOR Cell Proliferation

**Figure 4:**
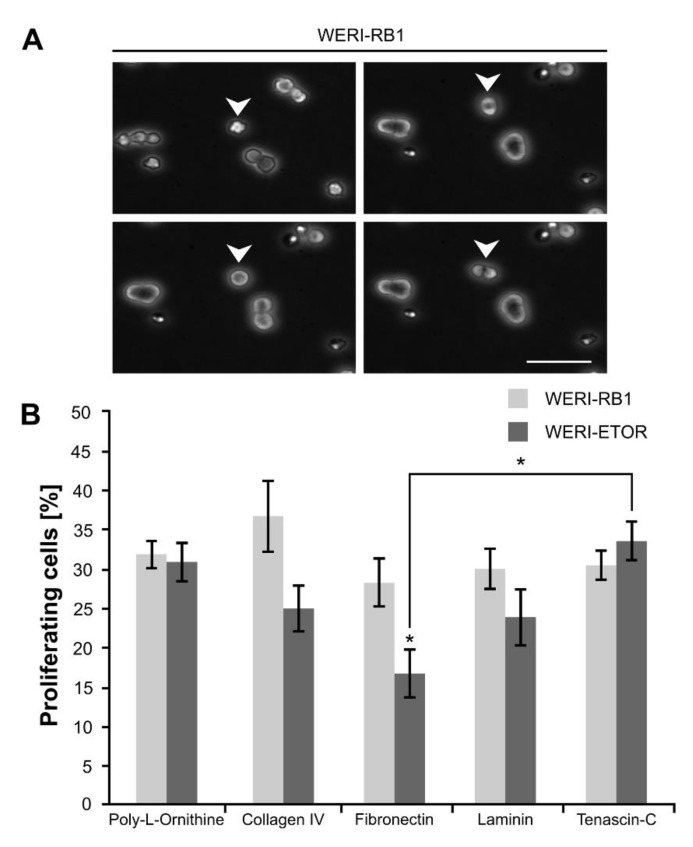
Proliferation analyses via time-lapse video microscopy of WERI-RB1 and WERI-ETOR cells cultivated on various ECM substrates. (**A**) Representative phase contrast images of proliferative WERI-RB1 cells documented via time-lapse video microscopy. White arrowheads indicate cell division. (**B**) The percentage of proliferative WERI-RB1 was comparable to the percentage of proliferative WERI-ETOR cells when cultivated on Poly-L-Ornithine (non-ECM control), Collagen IV, Laminin, and Tenascin-C. In contrast, on Fibronectin, a significant reduced percentage of proliferative WERI-ETOR cells was observed. Furthermore, regarding the WERI-ETOR cell line, a significant lower percentage of proliferative cells was found on Fibronectin compared to Tenascin-C. Values are shown as mean ± SEM. * *p* < 0.05; *n* = 5/group. Scale bar = 50 µm.

Additionally, we analyzed whether various ECM substrates influence the proliferation behavior of WERI-RB1 and WERI-ETOR cells. For this purpose, the number of daughter cells, generated by cell divisions over a period of 48 hours, was evaluated by time-lapse video microscopy (Figure 4A,B).

On Poly-L-Ornithine, no differences were found regarding the percentage of generated daughter cells in both cell lines (WERI-RB1: 31.91 ± 1.81%; WERI-ETOR: 30.93 ± 2.45%; *p* = 0.76). Also, a comparable number of cell divisions was observed on Collagen IV in WERI-RB1 (36.76 ± 4.54%) as well as in WERI-ETOR cells (25.02 ± 2.91%; *p* = 0.06). However, a trend towards a decreased cell division number was obvious in WERI-ETOR. Interestingly, a significant reduction in the number of cell divisions was seen for WERI-ETOR cells when cultivated on Fibronectin (WERI-RB1: 28.27 ± 2.99%; WERI-ETOR: 16.76 ± 3.04%; *p* = 0.03). On Laminin and Tenascin-C, no alteration in the percentage of generated daughter cells was noted in WERI-RB1 (Laminin: 30.04 ± 2.54%; Tenascin-C: 30.50 ± 1.89%) compared to WERI-ETOR cells (Laminin: 23.91 ± 3.58%; *p* = 0.2; Tenascin-C: 33.58 ± 2.42%; *p* = 0.34).

Most interestingly, when compared to Fibronectin coating, WERI-ETOR cells show a significantly increased number of cell divisions when cultivated on Tenascin-C (*p* = 0.01). The WERI-RB1 cells exhibited a similar fraction of proliferating cells on all investigated ECM substrates (*p* > 0.05).

**Figure 5:**
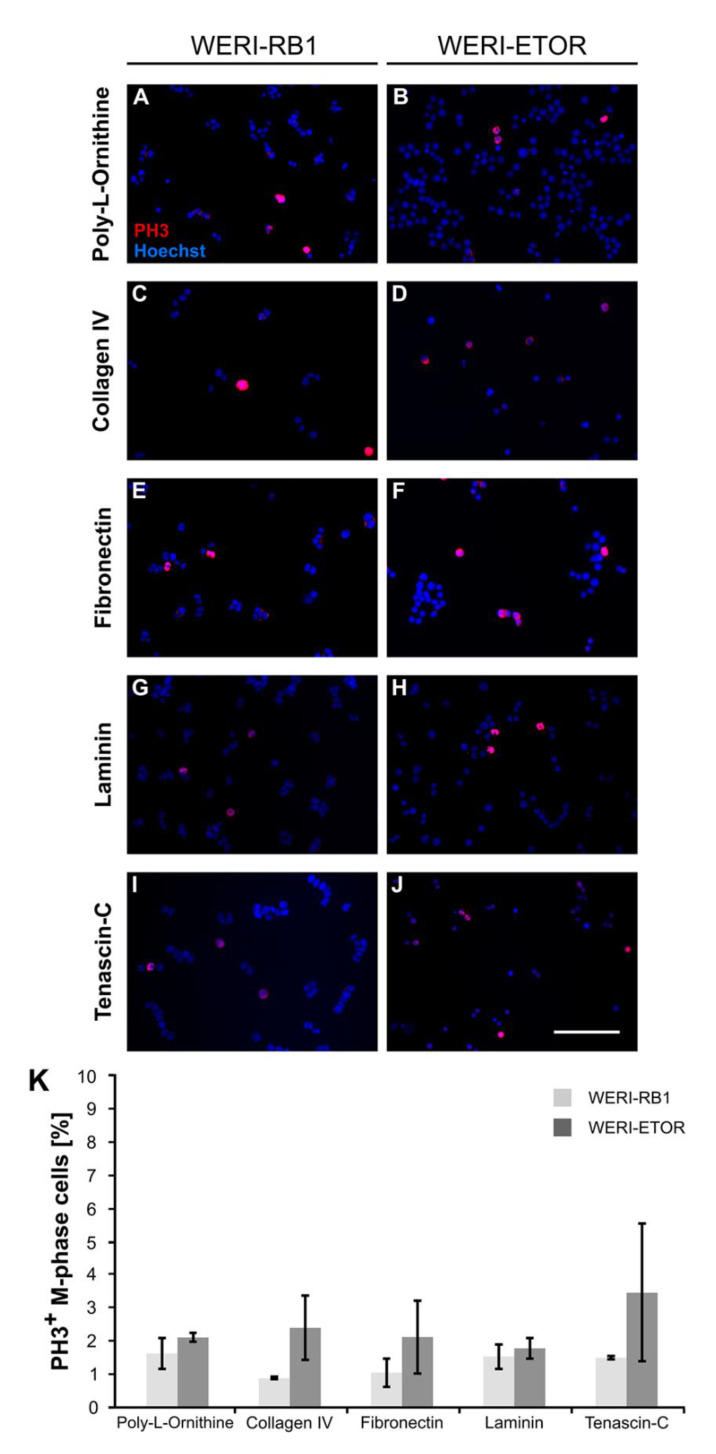
Immunocytochemical detection of PH3^+^ M-phase WERI-RB1 and WERI-ETOR cells cultivated on various ECM substrates. (**A–J**) Proliferative WERI-RB1 (**A,C,E,G,I**) and WERI-ETOR (**B,D,F,H,J**) M-phase cells were identified by PH3 immuno- (red) and nuclear Hoechst co-staining (blue). (**K**) In both cell lines, counts revealed that the percentage of M-phase cells was comparable when cultivated on Poly-L-Ornithine (non-ECM control), Collagen IV, Fibronectin, Laminin, and Tenascin-C. Values are shown as mean ± SEM. *n* = 3/group. Scale bar = 100 µm.

To evaluate the proliferation of both cell lines in more detail, cells were cultivated for 2 div, fixed and immunocytochemically stained with an anti-phospho Histone H3 (PH3) antibody (Figure 5A–K). Cell nuclei were counterstained with Hoechst and PH3^+^ M-phase (mitotic-phase) cells were counted.

On Poly-L-Ornithine, the number of M-phase^+^ WERI-ETOR cells (2.14 ± 0.13%) was comparable to the number of proliferative WERI-RB1 cells (1.66 ± 0.46%; *p* = 0.37). When cultivated on Collagen IV, a slightly increased number of PH3^+^ WERI-ETOR cells (2.43 ± 0.98%) was noted compared to WERI-RB1 cells (0.91 ± 0.04%; *p* < 0.05). Similar numbers of PH3^+^ M-phase cells were detected for both cell lines when cultivated on Fibronectin (WERI-RB1: 1.08 ± 0.43%; WERI-ETOR: 2.15 ± 1.09%; *p* = 0.41). The same tendency was observed for both cell lines cultured on Laminin (WERI-RB1: 1.57 ± 0.38%; WERI-ETOR: 1.81 ± 0.32%; *p* = 0.65) and Tenascin-C (WERI-RB1: 1.53 ± 0.05%; WERI-ETOR: 3.49 ± 2.07%; *p* = 0.40).

In summary, the different substrates appeared to exhibit no significant influence on the number of PH3^+^ M-phase WERI-RB1 and WERI-ETOR cells.

### 2.9. Cluster Formation Capacity of WERI-RB1 and WERI-ETOR Cells on Different ECM Substrates

**Figure 6:**
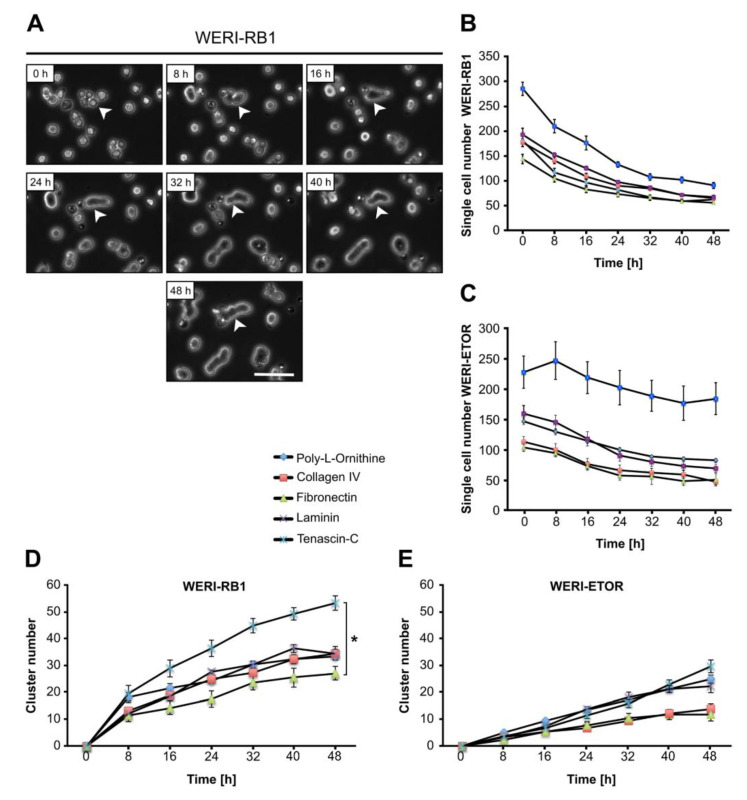
Single cell number and cell cluster formation analyses of WERI-RB1 and WERI-ETOR cells cultivated on various ECM substrates via time-lapse microscopy. (**A**) Representative phase contrast images of WERI-RB1 cell cluster formation (white arrowhead) as revealed by time-lapse video microscopy over 48 h. (**B,C**) The number of single WERI-RB1 and WERI-ETOR cells cultivated on Poly-L-Ornithine (non-ECM control), Collagen IV, Fibronectin, Laminin, and Tenascin-C decreases over time. (**D,E**) Over 48 h, the number of WERI-RB1 and WERI-ETOR cell clusters was increased when plated on various substrates. Importantly, at 48 h the WERI-RB1 cells displayed a significantly higher cluster number on Tenascin-C compared to Fibronectin. Data are shown as mean ± SEM. * *p* < 0.05; *n* = 5/group. Scale bar = 50 µm.

**Figure 7:**
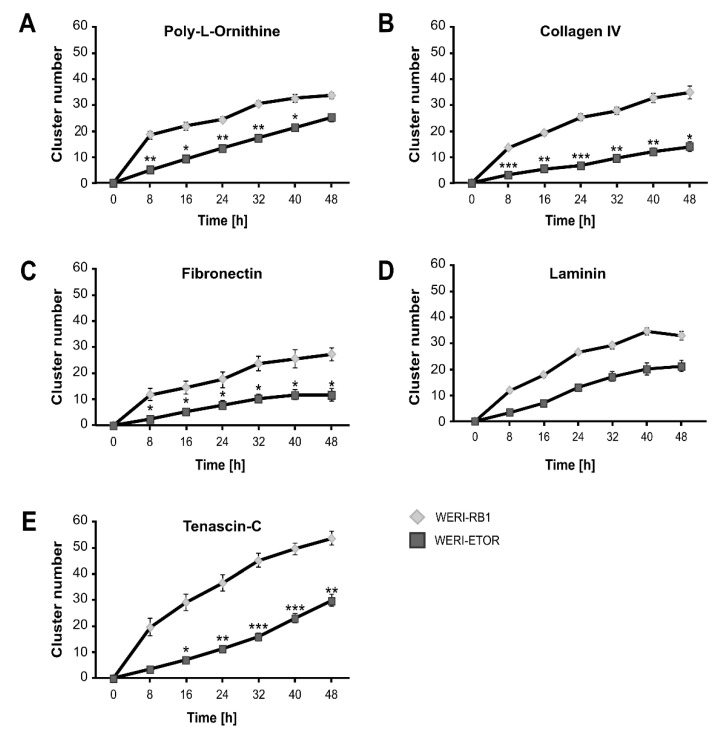
Cell cluster number of WERI-RB1 and WERI-ETOR cells cultivated on various ECM substrates via time-lapse microscopy. (**A**) In comparison to the WERI-RB1 cell line, WERI-ETOR showed a significantly decreased cluster number between 8 and 40 h when cultivated on Poly-L-Ornithine (non-ECM control). (**B**) On Collagen IV, WERI-ETOR cells displayed a lower cluster number from 8 to 48 h. (**C**) Also, on Fibronectin, WERI-ETOR cells displayed a reduced cluster number from 8 to 48 h. (**D**) In contrast, both RB cell lines exhibited a similar cluster number on Laminin. (**E**) On Tenascin-C, WERI-ETOR showed a significantly reduced cluster number from 16 to 48 h. Data are shown as mean ± SEM. * *p* < 0.05; ** *p* < 0.01; *** *p* < 0.001; *n* = 3/group.

In suspension, both WERI cell lines display cluster and chain formation. To investigate cell cluster formation in dependence of various ECM substrates, dissociated cells were analyzed via time-lapse video microscopy. Therefore, the total number of single cells and cell clusters, defined as ≥ 3 cells, was counted after plating for 0, 8, 16, 24, 32, 40, and 48 hours (Figure 6 and Figure 7).

Attached to various substrates, the number of dissociated single WERI-RB1 and WERI-ETOR cells decreased with ongoing time continuously (Figure 6A–C), whereas the number of clusters increased steadily (Figure 6D–E). This general behavior was observed for both cell lines on each ECM substrate. Regarding the cluster number of the WERI-RB1, only Tenascin-C and Fibronectin exhibited a significant impact on cluster formation at 48 h (Figure 6D). At 48 h, an increased cluster rate was observed on Tenascin-C (*p* = 0.02), when compared to Fibronectin. WERI-ETOR cells displayed similar cluster rates on all used substrates (*p* > 0.05; Figure 6E). Taken together, Tenascin-C significantly promoted cluster formation, while Fibronectin decreased cluster formation of WERI-RB1, but not WERI-ETOR cells.

Next, the cluster rate of WERI-RB1 and WERI-ETOR cells was analyzed in dependence of the ECM substrates (Figure 7A–E).

Overall, the number of clusters was lower in the WERI-ETOR than in the WERI-RB1 cell line. WERI-ETOR cells, cultivated on Poly-L-Ornithine, displayed a significantly decreased cluster formation capacity from 8 to 40 h (*p* < 0.05; Figure 7A). Also, on Collagen IV and Fibronectin, the number of WERI-ETOR cell clusters was significantly lower between 8 and 48 h (both *p* < 0.05; Figure 7B–C). In contrast, no difference in the cluster formation rate was found for WERI-RB1 and WERI-ETOR cells when cultivated on Laminin (*p* > 0.05; Figure 7D). On Tenascin-C, a decreased cluster number was observed for WERI-ETOR cells between 16 and 48 h (*p* < 0.05; Figure 7E).

Collectively, these findings indicate that WERI-ETOR cells exhibited a lower cluster forming capacity than WERI-RB1 cells when cultivated on Poly-L-Ornithine and the ECM substrates Collagen IV, Fibronectin, and Tenascin-C.

## 3. Discussion

The mechanisms that contribute to the development of chemoresistance are extremely complex. In recent decades, the ECM, with its diverse functions and complex regulatory influences, has increasingly become a focus of oncological research. The tumor microenvironment, which consists of the tumor´s vascular system, the surrounding connective tissue, infiltrating immune cells, and the ECM are important components in the development of chemotherapy resistance, but there is still a lot more to explore [46]. Especially the composition, organization as well as post-translational modifications of the tumor ECM are decisive factors that influence tumor development, growth, angiogenesis, metastasis, and also chemosensitivity [19,20,21,22,23,24,47].

### 3.1. Expression of CSPGs in RB Cells

Changes of CSPGs have been seen in several solid tumors so far [48,49,50,51,52,53,54,55]. However, currently we do not know the function of CSPG alterations in solid tumors. In our study, we noted a downregulation of the CSPGs *BCAN*, *NCAN,* and *VCAN*, but no different regulation of *ACAN* mRNA level in WERI-ETOR. As revealed for the WERI-ETOR cell line, our analyses showed a significantly lower expression level of *BCAN* in Etoposide resistant Y79 cells and a comparable expression level of *ACAN* in both Y79 cell lines. However, in contrast to the observed downregulation of *NCAN* and *VCAN* in the WERI-ETOR cell line, mRNA expression of these proteoglycans was highly upregulated in the Etoposide resistant Y79 cell line. Therefore, we assume that a common alteration in RB seems to be responsible for the significantly lower expression level of *BCAN* in Etoposide resistant RB cells and comparable level of *ACAN* in parental and resistant RB cells. In contrast, alterations of *NCAN* and *VCAN* seem to be more cell line specific. An up-regulation of CSPGs in tumors and their association with metastasis and malignancy has been demonstrated in different cancer types, such as glioma and lung carcinoma [48,49,50,51,52,53,54,55]. All of this knowledge seems contrary to our findings regarding *BCAN* and *ACAN*, since it displays the situation in adult tumors. In our study, we investigated a childhood tumor. Considering retinal development, this may explain the meaning of CSPG downregulation in RB. In CNS neurons, glia as well as neural stem cells highly express CSPGs, such as Aggrecan, Brevican, Neurocan, and Versican [56,57]. For all of these CSPGs, a downregulated expression pattern was demonstrated during retinal development [58,59]. Therefore, the observed downregulation of *BCAN*, *NCAN,* and *VCAN* in Etoposide resistant RB cells contrasts with the expression pattern in adult cancer but might represent the expression pattern in childhood tumors. On the other hand side, *BCAN* expression has been seen during retinal development as well as in human pluripotent stem cell derived retinal organoids [60]. In this regard, the downregulation of *BCAN* might be associated with stem cell characteristics in resistant RB cells.

As revealed for the WERI-ETOR cell line, our analyses found a significantly lower expression level of *BCAN* in Etoposide resistant Y79 cells and a comparable expression level for *ACAN* in both Y79 cell lines. However, in contrast to the observed downregulation of *NCAN* and *VCAN* in the WERI-ETOR cell line, mRNA expression of both proteoglycans was highly upregulated in the resistant Y79 cell line. These expression differences between the two resistant cell lines might point to the fact that expression changes dependent on the intrinsic cellular response of the cell line, rather than Etoposide chemoresistance. As already mentioned above, the prominent upregulation of *NCAN* and *VCAN* in resistant Y79 cells might also correlate with their role in tumor growth and invasion as it has been demonstrated in glioma and in lung carcinoma [48,49,52,53,54,55].

### 3.2. Expression of Glycoproteins and Collagen IV in RB Cells

The interplay between tumor cells and stroma has been suggested to play a key role in tumor progression and sensitivity to chemotherapy [61]. Within our study, we observed a downregulation of *COL4A1* in both Etoposide resistant cell lines. As an important component of basement membranes, Collagen IV plays a key role in various tumor types. It stimulates proliferation and migration of pancreatic cancer cells [62]. Additionally, the interaction of pancreatic cancer cells with Collagen IV decreased their sensitivity to cytotoxic drugs and promoted cell proliferation [63]. Furthermore, Collagen IV silencing by small-interfering RNA decreases hepatic metastases formation [64]. In the human retina, it can be found in the Bruch`s membrane of the retinal pigment epithelium [65]. Collagen IV is in addition expressed by blood vessels, where it may play a role in neovascularization, blood vessel maturation, and metastasis in RB tumors [66,67]. We observed a *COL4A1* downregulation in both Etoposide resistant RB cell lines, which seems to be in contrast with the observed upregulation and functional relevance in various tumor types. However, Fang et al. reported that Collagens, including Collagen IV, can be a double-edged sword in tumor progression, both inhibiting and promoting tumor progression at various stages of cancer development [68]. Collagen degradation, e.g. shown in the colorectal adenocarcinoma cell line HCT-8, is of importance for leaving space for ECM remodeling towards tumor metastasis. Skubitz and colleagues demonstrated that Y79 RB cells exhibit a specific adhesion to Fibronectin, but not to Collagen and Laminin [69]. Therefore, our observed downregulation of *COL4A1* in both Etoposide resistant RB cell lines might reflect a functional relevance in the regulation of cellular adhesion due to ECM remodeling after Collagen IV degradation. Recent studies demonstrated the posttranslational alteration of collagens by enzymes like Lysyl oxidases, Focal adhesion kinase, and Prolyl-4-hydroxylases in cancer drug resistance [70,71,72,73,74,75,76,77]. Here, resistance seems to be accomplished by an increased stability of modified helical collagen. Therefore, further analyses of posttranslational collagen alterations should be performed in RB chemotherapy resistance investigations.

Expression of *FN1* was also reduced in WERI-ETOR, while resistant Y79 cells displayed only a trend towards a reduced but comparable expression level in comparison to the sensitive Y79 cells. Tumor cell migration, invasion and metastasis are essentially influenced by Fibronectin–Integrin interaction and alterations in the tumor microenvironment [78,79,80,81,82]. Especially in breast cancer, increased amounts of Fibronectin activate the FAK/ILK/ERK/PIK/NK-κB signaling pathway and hereby mediate an up-regulation of MMP-2 and -9 [83]. Taken together, ECM alterations are seen in tumor metastasis, but not in RB chemotherapy resistance.

*LAMA1* expression was downregulated in resistant WERI-ETOR. However, in contrast to the findings in the resistant WERI-ETOR cell line, expression of *LAMA1* was significantly upregulated in the resistant Y79 cell line.

Therefore, our data suggest that *COL4A1* and probably also *FN1* are associated with chemotherapy resistance in RB, while *LAMA1* alterations seem to be RB cell line specific.

### 3.3. Expression of Tenascin-C and Tenascin-R in RB Cells

The importance of Tenascins in retina development and diseases has been postulated [84,85]. In addition, glioma malignancy grade, and poor prognosis correspond with a high expression of Tenascin-C [86,87,88]. Furthermore, various studies explored Tenascin-C as trigger for cancer drug resistance [89,90,91,92]. A study on human colon carcinoma tissue demonstrated a link between Tenascin-C upregulation and cancer cell invasion [93].

In our study, we noted a significant downregulation of *TNC* expression in WERI-ETOR cells. While *TNC* expression was comparable in the sensitive and resistant Y79 cell lines. Analysis on protein level, on the other side, did not show differences in the Tenascin-C concentration in WERI-RB1 and WERI-ETOR cells. Additionally, a higher cell death rate was observed for WERI-ETOR on Tenascin-C coating. Therefore, we assume, that expression and protein level of Tenascin-C seem to be similar in a chemotherapy resistant and sensitive situation in RB but may lead to malignancy and chemotherapy resistance through unknown posttranslational alterations in this matrix protein and/or an isoform-dependent regulation.

In the present study, we described a massive downregulation of *TNR* in WERI-ETOR and resistant Y79 RB cells. Inhibiting properties of Tenascin-R were described in regard to adhesion of mesenchymal and neural cells on Fibronectin, whereas the role of Tenascin-R has barely been investigated in cancer [87,94,95]. Our data indicate a statistically significantly lower level of clustering for WERI-ETOR on Fibronectin in comparison to WERI-RB1. This might be triggered by downregulation of *TNR* expression.

### 3.4. Expression of MMPs and TIMPs in RB Cells

In the present study, we noted a dramatic downregulation of *MMP2*, *MMP7,* as well as *MMP9* expression in WERI-ETOR. A statistically significant downregulation of *MMP2* expression was confirmed in resistant Y79 cells. While analysis on protein level for pro- and active-MMP-2 in WERI-RB1 and WERI-ETOR did not show any striking differences. Previous studies by other research groups, on the other hand, observed a high expression of MMP-2 and MMP-9 in correlation with a higher clinical grading of RB tissue [96,97]. Also, inhibition of MMP-2 and MMP-9 led to a decreased cellular migration and angiogenesis in in vitro models of RB [98]. In contrast, high levels of *MMP2* were found to be associated with a better chemotherapy response in ovarian cancer tissue [99]. Therefore, downregulation of *MMP2* may lead to chemotherapy resistance in RB via an unknown mechanism. Nevertheless, on protein level MMP-2 was not reduced in resistant WERI-ETOR cells, which should be investigated further.

Aditi and colleagues investigated MMP-2, MMP-9, TIMP-1, and TIMP-2 expression in RB tissue via immunohistochemical staining and immunoblotting [97]. Upregulation of MMPs and TIMPs correlated with a higher clinical RB malignancy scoring and therefore with metastasis. Contradictory to these data, our results did not show differences in *TIMP1* expression between both WERI cell lines, but a downregulation of *TIMP2* expression in chemotherapy resistant WERI-ETOR cells. Expression analysis of Y79 confirmed the downregulation of *TIMP2* but revealed an upregulation of *TIMP1* in resistant Y79 cells. On the one hand, different reports revealed the complexity of the MMP and TIMP interaction on ECM in cancer. A Bcl-2 mediated anti-apoptotic effect was seen for TIMPs in tumor cells [100]. On the other hand, TIMPs inhibited tumor invasion and metastasis [101]. TIMP-2 upregulation was linked to metastasis via MMP-2 inactivation in nasopharyngeal carcinoma [102]. Additionally, a high ratio of MMP-9 to TIMP-2, implicating that a higher TIMP-2 level leads to drug resistance, was beneficial for the treatment of metastatic renal cell carcinoma [103]. Furthermore, higher tissue levels of TIMP-2 marked a favorable prognosis in ovarian cancer [104]. In this line, therapeutic upregulation of TIMP-2 reduced the invasiveness of a metastatic breast cancer cell line [105]. TIMP-2 might have the same function in RB. In summary, the interplay between MMPs and TIMPs seems to be rather complex in RB metastasis, but *TIMP-2* downregulation might lead to metastasis and chemotherapy resistance in RB.

### 3.5. Integrin Expression in RB Cells

In our study, we observed a downregulation of *ITGA4*, *ITGA5,* and *ITGB1* expression in WERI-ETOR, but only *ITGA5* expression downregulation was confirmed in resistant Y79 cells. Interestingly, α5-Integrin has been described as a potential suppressor for tumor metastasis in a non-metastatic breast cancer cell line, whereas α6-Integrin had opposed characteristics in a metastatic breast cancer cell line and may promote metastasis [106]. In addition, a Fibronectin-dependent enhanced expression of α5β1 Integrin is associated with ovarian cancer metastasis [107]. Some specific Integrins were also described as tumor suppressors, especially α5 Integrin has been reported to inhibit tumor cell growth through their effects on cell-cycle-regulating proteins and proteins responsible for DNA repair [106,108]. Therefore, it could be possible that the observed downregulation of α5 Integrin in resistant RB cells might be associated with chemotherapy resistance by promoting cell cycle progression and by enhancing the repair of DNA double-strand breaks caused by various chemotherapeutic agents including Etoposide. Also, an upregulation of α5β1 compromised p53 induced chemotherapy sensitivity in high grade glioma [109]. However, α5β1 Integrin promotes invasive tumor protrusions by promoting joint Integrin/Receptor tyrosine kinase signaling [110,111]. In summary, we assume that downregulation of *ITGA5* expression in RB might be associated with a higher metastasis rate and chemotherapy resistance. However, this finding needs to be investigated in more detail.

### 3.6. Expression of RPTPβ/ζ in RB Cells

During adolescence, the *RPTPβ/ζ* isoform *Phosphacan* is expressed in Müller cells, while *RPTPβ/ζ_long_* is barely expressed in the adult retina [58,85]. In our study, using a set of various primers, we analyzed *RPTPβ/ζ_long_*, *RPTPβ/*ζ_short_, and *Phosphacan* in WERI-RB1. In general, the *RPTPβ/*ζ isoforms were, if ever, only slightly expressed in WERI-ETOR cells. As a functional binding partner of the vascular endothelial growth factor, *RPTPβ/*ζ is postulated to be an angiogenesis promoting factor, a potential hallmark for metastasis [112]. Interestingly, in concordance with our results, Diamantopoulou and colleagues demonstrated that a loss of *RPTPβ/*ζ initiates epithelial-to-mesenchymal transition and leads to metastasis in prostate cancer [113]. Therefore, our observed downregulation or loss of *RPTPβ/*ζ isoforms may indicate an epithelial-to-mesenchymal transition towards a chemotherapy resistance in RB.

Phosphacan/RPTPβ/ζ is an important and well described binding partner and receptor of the ECM glycoprotein Tenascin-C [43,44]. Furthermore, Tenascin-C directly interacts with the CSPG Neurocan [114] and Integrin receptors [115]. Additionally, it is cleaved by various MMPs [116]. Due to these facts, it was interesting that we observed a prominent downregulation of all RPTPβ/ζ isoforms in resistant WERI-ETOR cells. This result could also point to reduced interacting ECM network between Tenascin-C and the CSPGs Phosphacan and Neurocan, the RPTPβ/ζ receptor as well as MMPs. However, there is still a lot to explore and discover, before understanding the interaction of the ECM network in RB.

### 3.7. ECM Influence on the Apoptotic Rate of RB Cells

In this study, we investigated the rate of apoptotic RB cells by cleaved Caspase 3 immunostaining. A significantly higher apoptotic rate was present in WERI-RB1 cultivated on Fibronectin in comparison to all other tested substrates. Interestingly, a significantly increased apoptotic cell number was seen in WERI-ETOR in comparison to WERI-RB1 cells after cultivation on Tenascin-C. However, one should consider that the intrinsic apoptosis rate of WERI-ETOR is higher, as seen on the Poly-Ornithine condition. Different studies verified a correlation between Tenascin-C expression and prognosis in different cancer types, like chondrosarcoma as well as prostate and breast cancer [91,117,118,119]. On the one hand, a promoting effect on apoptosis was reported for Fibronectin in prostate cancer cells [120]. On the other hand, Fibronectin obtained an anti-apoptotic effect via Bcl-2 (B-cell lymphoma) in cancer [121]. Therefore, especially Fibronectin alterations might lead to different malignancy and chemotherapy resistance in RB.

### 3.8. ECM Influence on the Proliferation Rate of RB Cells

In the present study, we investigated the effect of Poly-L-Ornithine, Collagen IV, Fibronectin, Laminin, and Tenascin-C on WERI-RB1 and WERI-ETOR proliferation. A significantly lower proliferation of WERI-ETOR on Fibronectin in comparison to Tenascin-C coating was only detected by time-lapse video microscopy, but not by phospho Histone H3 and Hoechst co-staining. Previous studies demonstrated a positive effect of Fibronectin and Collagen IV on lens epithelial cell proliferation [122]. In accordance with these findings, Illario and colleagues noted an increased mitosis rate for thyroid cells cultivated on Fibronectin, including cell adhesion and spreading [123]. Interestingly, Orend et al. observed an inhibitory effect of Tenascin-C on Fibronectin promoted proliferation in fibroblasts [124]. In sum, our data revealed a higher proliferation rate of WERI-ETOR on Tenascin-C in comparison to Fibronectin, which further supports the idea of a switch from a Fibronectin dominated chemotherapy sensitive ECM environment to a Tenascin-C dominated chemotherapy resistant ECM environment.

### 3.9. ECM Influence on Cluster Formation of RB Cells

Circulating tumor cell clusters were observed in the peripheral blood of colorectal cancer patients [125]. The same effects were seen in circulating breast tumor cells [126]. In contrast to metastatic data, our results indicate a higher number of clusters in WERI-RB1. This phenomenon might be explained by the different nature of RB tumors, including RB protein loss, in comparison to previously mentioned, mostly carcinoma, tumors. Hence this should be further investigated. Interestingly, WERI-ETOR cells displayed a reduced cluster formation capacity on Tenascin-C. Indeed, Tenascin-C can exhibit repulsive properties, which may lead to a reduced aggregate formation. In this regard, a counterbalancing anti-adhesive effect of Tenascin-C through Fibronectin expression was reported in endothelial cells [127].

### 3.10. Limitations and Future Perspectives of the Study

The aim of the present study was to explore the composition of the ECM and its alteration in RB chemotherapy resistance. In our study, most experiments have been performed in the RB cell lines WERI-RB1 and WERI-ETOR. We performed *in vitro* proliferation, cell death and adhesion analyses in both cell lines. Additionally, both cell lines were tested in regard to the mRNA expression of various ECM molecules as well as several modulating enzymes and receptors. Here, we found mRNA differences between WERI-RB1 and WERI-ETOR, which might display the situation in human RB. Also, exemplary protein analyses via Western blot for TNC and MMP-2 were performed in WERI-RB1 and WERI-ETOR cell lines. As we found no differences for these two proteins and other proteins were not tested so far, future analyses should uncover possible protein alterations in both RB cell lines. In order to evaluate mRNA expression in two independent cell lines, analyses regarding the expression of the CSPGs *ACAN*, *BCAN*, *NCAN,* and *VCAN*, the glycoproteins *LAMA1*, *FN1*, and *TNC* and *TNR*, *MMP2*, *MMP7*, *MMP9*, *TIMP1*, and *TIMP2* and the integrin receptor subunits *ITGA4*, *ITGA5,* as well as *ITGB1* were also performed in Y79 and resistant Y79 RB cells.

A study limitation was that many of the mRNA results need to be confirmed by Western blotting. In a follow up project, experiments should focus on protein levels of both Y79 cell lines. A further limitation of this study was that most data were only from one cell line and its subclone (WERI-RB1 and WERI-ETOR). Therefore, future studies should also focus on *in vitro* analyses in Y79 cells.

## 4. Materials and Methods

### 4.1. Cultivation and Authentication Verification of Human RB Cell Lines

The Etoposide sensitive and resistant RB cell lines WERI-RB1 and Y79 were cultured as described previously [36,37,38]. Briefly, cells were cultured in T75 flasks (Greiner, Kreuzmünster, Austria) in Dulbecco’s Modified Eagle’s Media (DMEM) supplemented with 15% fetal calf serum (FCS), 1% penicillin and streptomycin, 4 mM L-glutamine, 50 μM β-mercaptoethanol and 10 μg/mL insulin (all Sigma-Aldrich, St. Louis, MO, USA) at 37 °C and 10% CO_2_.

Identities of all RB cell lines were verified by DNA fingerprinting and profiling using eight different and highly polymorphic short tandem repeat (STR) loci (Leibniz-Institute DSMZ GmbH, Braunschweig). Generated STR profiles of cell lines showed a full match of the respective parental reference STR profiles as indicated by a search of the cell bank databases ATCC (Manassas, VA, USA), JCRB (Osaka, Japan) and RIKEN (Ibaraki, Japan), KCLB (Seoul, Korea), as well as DSMZ (Braunschweig, Germany). Also, purity of cell lines was proven by analyses of mitochondrial DNA sequences from mouse, rat or Chinese and Syrian hamster cells. Analyses, with a detection limit of 1:10^5^, revealed the absence of any mitochondrial sequences from foreign species. Hence, the origin and purity of the human RB cell lines was determined.

### 4.2. Cultivation of the Human Glioblastoma Cell Line U-251-MG and Purification of Human Tenascin-C

For the purification of human Tenascin-C, the cell line U-251-MG was cultured in T250 flasks (Thermo Fischer Scientific, Breda, Netherlands) in Minimum Essential Medium α with GlutaMAX I (α-MEM; Thermo Fischer Scientific) supplemented with 10% FCS and 0.1% gentamycin (Roth, Karlsruhe, Germany) at 37 °C and 6% CO_2_. After 7 days, the supernatant was collected. For Tenascin-C purification, affinity chromatography was performed using the monoclonal antibodies 608 and 19H12 as described previously [86].

### 4.3. Cell Proliferation and Cluster Formation Analyses Via Time-Lapse Video Microscopy

In order to analyze the proliferation and cluster formation behavior of the WERI cells on different ECM substrates, WERI-RB1 and WERI-ETOR cells were dissociated in 1 mL Trypsin/EDTA (Thermo Fisher Scientific) at 37 °C for 5 min. Thereafter, enzymatic dissociation of cells was stopped using an equal amount of Ovomucoid containing 1 mg/mL soybean trypsin inhibitor in L-15 medium (both Sigma-Aldrich), 40 µg/mL DNase I (Worthington, Lakewood, USA), and 50 µg/mL bovine serum albumin (BSA; Sigma-Aldrich). Following resuspension in fresh serum-free culture medium, cell numbers were determined in a Neubauer counting chamber (Brand, Wertheim, Germany). 24-well-dishes (Thermo Fisher Scientific) were pre-coated with Poly-L-Ornithine (10 μg/mL; Sigma-Aldrich) in H_2_O. Next, wells were coated with ECM substrates, namely human Fibronectin (10 μg/mL; Corning Inc., Wiesbaden, Germany), mouse Laminin (10 μg/mL; Corning Inc.), human Collagen IV (10 μg/mL; Sigma-Aldrich), or human Tenascin-C (50 μg/mL) in 1 × PBS. 3 × 10^4^ dissociated cells were seeded in 500 μL serum free culture medium per well. Cells were documented via time-lapse video microscopy (Axiovert 200M; Zeiss, Jena, Germany) in a heated chamber at 37 °C and 10% CO_2_ for 48 h. An image of each well was recorded every 8 min and combined for a time-lapse video. At 0, 8, 16, 24, 32, 40, and 48 hours after plating, we counted the number of single cells as well as cell clusters, defined as 3 or more cells.

### 4.4. RNA Isolation and cDNA Synthesis

To obtain mRNA, 5 × 10^6^–1 × 10^7^ Etoposide resistant and sensitive WERI, Etoposide resistant and sensitive and Y79 RB cells or U-251-MG cells were snap frozen in liquid nitrogen. Total RNA extraction was performed with the Gene Elute Mammalian Total RNA Miniprep Kit (Sigma-Aldrich) following the manufacturer’s instructions. Purity and concentration of RNA was analyzed using a BioSpectrometer (Eppendorf, Hamburg, Germany). For cDNA synthesis, 1 µg RNA was reverse-transcripted with the First Strand cDNA Synthesis Kit using random hexamer primers (Thermo Fisher Scientific).

### 4.5. Quantitative Real-Time PCR Analyses (RT-qPCR)

RT-qPCR analyses were performed using the FastStart essential DNA green master mix in a Light Cycler 96 (Roche Applied Science, Mannheim, Germany). Reaction conditions were as follows: 10 min at 95 °C (pre-incubation), followed by 10 sec at 95 °C, 30 sec at 60 °C, and 10 sec at 72 °C for 45 cycles (amplification), 10 sec at 95 °C, 60 sec at 65 °C, and 1 sec at 97 °C (melting curve analyses) and 30 sec at 37 °C (cool down period). Primer pairs for RT-qPCR (Sigma-Aldrich) were designed using the ProbeFinder Assay Design Center (Roche Applied Science; Table 1). Primer efficiency of each primer was calculated based on a cDNA dilution series of 5 ng to 125 ng. The housekeeping genes *β-Actin (ACTB), Glyceraldehyde 3-phosphate dehydrogenase (GAPDH)*, and *S18* (*RBPS18*) were used for normalization and relative quantification.

### 4.6. Semi-Quantitative Reverse-Transcription PCR Analyses (RT-PCR)

By combining different primer pairs, modified according to Norman et al., unique *RPTPβ/ζ (PTPRZ1)* isoforms were analyzed via semi-quantitative RT-PCR (Table 2) [45]. RT-PCR using CP and EC primer pairs was performed with Taq DNA Polymerase (Sigma-Aldrich). For the amplification of long products with TEC primers, the GoTaq Long PCR Master Mix (Promega, Mannheim, Germany) was used. RT-PCR was performed in a Mastercycler Gradient (Eppendorf, Hamburg, Germany). RT-PCR reaction conditions for CP and EC primers were as follows: 5 min at 94 °C (pre-denaturation), followed by 28 cycles (*ACTB*) to 38 cycles (*PTPRZ1* isoforms) of 30 sec at 94 °C (denaturation), 30 sec at 60 °C (annealing), and 30 sec at 72 °C (elongation). For RT-PCR using TEC primers the following conditions were used: 2 min at 94°C (pre-denaturation), 40 cycles of 30 sec at 94 °C (denaturation), 30 sec at 60 °C (annealing), and 3 min at 65 °C (elongation). The products were separated in 1.5% agarose gels.

### 4.7. Immunocytochemistry

For immunocytochemical staining, 15 × 10^3^ dissociated cells were seeded on Poly-L-Ornithine pre-coated 4-well-dishes (3 wells/condition; Thermo Fisher Scientific) and incubated for 2 div. After cultivation, cells were washed and blocked with prewarmed Krebs´ solution-Ringer`s solution-HEPES buffer (KRH/A; 125 mM NaCl, 4.8 mM KCl, 1.3 mM CaCl_2_·2 H_2_O, 1.2 mM MgSO_4_·7 H_2_O, 1.2 mM KH_2_PO_4_, 5.6 mM D-glucose, 25 mM HEPES and 0.1% BSA). After washing twice in KRH, cells were fixed with 4% paraformaldehyde (PFA) in 1x PBS at room temperature (RT) for 10 min and then washed twice in PBT1 (1x PBS, 0.1% Triton-X-100, 0.1% BSA). The cells were stained using anti-phospho Histone H3 (proliferative M-phase cells; PH3; rabbit, 1:100, Merck Millipore, Darmstadt, Germany) and anti-cleaved Caspase 3 antibodies (apoptotic cells; rabbit, 1:200, Sigma-Aldrich). To visualize F-Actin (Sigma-Aldrich), Phalloidin staining (1:100) was performed. Therefore, primary antibody/Phalloidin was diluted in PBT1 and this solution was applied to the cells. Following incubation for at RT 1 h, cells were washed twice with PBS/A (1× PBS, 0.1% BSA). Species-specific Cy/Cy3-coupled secondary antibodies (all Dianova GmbH, Hamburg, Germany), diluted 1:300 in PBS/A, were applied for at RT 1 h. To stain cell nuclei, the secondary antibody solution was supplemented with Hoechst (1:100.000; Hoechst 33258, Sigma-Aldrich). After two washing steps with 1× PBS, cells were mounted in 1×PBS/glycerol and covered with a glass coverslip. Stained cells were documented via an inverted fluorescence microscope (Axioplan 2; Zeiss). For each condition, 3 images/well were taken with 300–500 cells/visual field. For each staining and condition at least 3 independent experiments were performed. The counted cells were cultivated in 3 wells of a 4-well-dish with a negative control.

### 4.8. Protein Lysis and Western Blot

WERI-RB1, WERI-ETOR and U-251-MG cells (5 × 10^6^–1 × 10^7^) were homogenized in 100 μL lysis buffer (10 mM Tris base, 1 mM EDTA, 0.5 mM EGTA, 1% (*v/v*) Triton-X-100, 0.1% (*v/v*) SDS, 0.1% (*v/v*) sodium deoxycholate, 140 mM NaCl) on ice for 1 h. Afterwards, proteins were centrifuged at 14,000× g for 10 min at 4 °C. The Pierce^™^ BCA Protein Assay Reagent kit (Thermo Fisher Scientific) was used according to manufacturer’s instructions to determine the protein concentration in the supernatant. Protein samples (20 μg) were diluted in 4x loading buffer (250 mM Tris base, 40% glycerin, 20% β-mercaptoethanol, 20% SDS and 0.03 mM bromophenol blue) and denaturized at 95 °C for 5 min. Proteins were separated using 4–10% polyacrylamide gradient gels. By Western blotting, proteins were transferred to polyvinylidene difluoride (PVDF) membranes (Roth, Karlsruhe, Germany). Membranes were blocked in TBST blocking solution (5% *w/v* milk powder in Tris-buffered saline (TBS) and 0.05% Tween-20) at RT for 1 h and then incubated with primary antibody in blocking solution overnight at 4 °C. In order to detect Tenascin-C, a polyclonal anti-rabbit antibody was used in a dilution of 1:5,000 [128]. MMP-2 protein was analyzed by a rabbit antibody (H-76, 1:1,000, Santa Cruz Biotechnology, CA, USA). The housekeeping protein α-Tubulin was detected using a monoclonal anti-mouse antibody (DM1A, 1:10,000, Sigma-Aldrich). After antibody incubation, membranes were washed in TBST for 15 min and incubated with species-specific goat horseradish peroxidase (HRP) coupled secondary antibodies (1:5,000–1:10,000, Dianova GmbH) in blocking solution for 1 h at RT. Afterwards, membranes were washed in TBST for 10 min and TBS for 15 min at RT. To detect protein bands, the two solutions of the SuperSignal^™^ West Pico chemiluminescence kit (Thermo Fisher Scientific) were mixed 1:1 and added to the PVDF membrane. After incubation at RT for 5 min, chemiluminescence was detected and documented in a detection chamber (MicroChemi Chemilumiscence Reader, Biostep, Burkhardtsdorf, Germany). The intensity of TNC and pro- and active-MMP-2 protein bands was measured using TotalLab Quant software (TotalLab, Newcastle upon Tyne, UK) and normalized against the α-tubulin band intensity.

### 4.9. Statistical Analyses

Data of RT-qPCR are presented as median ± quartile + maximum/minimum and were analyzed by the pairwise fixed reallocation and randomization test (REST 2009 released Qiagen, [129]). RT-PCR data are shown as mean ± standard deviation (SD). Immunocytochemical, time-lapse video microscopy and Western blot data are presented as mean ± standard error mean (SEM). These data were analyzed using Student`s *t-test* for pairwise or ANOVA for multiple comparisons followed by Scheffés post-hoc test (Statistica version 12; Dell, Tulsa, OK, USA). For all statistical analyses, values of *p* < 0.05 were considered significant.

## 5. Conclusions

The aim of the study was to evaluate the expression and potential role of the ECM in RB chemotherapy resistance. Collectively, our data present information regarding ECM and interacting molecules in a chemotherapy resistance model of RB. We assume that the differential ECM expression in the Etoposide resistant compared to the Etoposide sensitive RB cell lines reflects a role of various ECM components in RB cell adhesion, proliferation as well as in chemotherapy resistance. Still, a lot work has to be done, before understanding the whole picture of ECM function and interplay with its enzymes in RB chemotherapy resistance.

## Figures and Tables

**Table 1:** List of primer pairs used for RT-qPCR analyses of RB cell lines. For relative quantification of mRNA levels, *β-Actin (ACTB), Glyceraldehyde 3-phosphate dehydrogenase (GAPDH)*, and *S18* (*RBPS18*) served as housekeeping genes. For each gene, the primer sequence and predicted amplicon size is indicated. Abbreviations: bp—base pairs, for—forward, rev—reverse.

Primer Name	Gene	Primer Sequence	Accession Number	Product Size (bp)
*Aggrecan* for	*ACAN*	tcaccagtgaggacctcgt	NM_001135.3	84
*Aggrecan* rev		ggcggtagtggaagacgac	NM_013227.3	
*β-Actin* for	*ACTB*	ccaaccgcgagaagatga	NM_001101.3	97
*β-Actin* rev		ccagaggcgtacagggatag		
*Brevican* for	*BCAN*	tggaagctccactccagaa	NM_021948.4	76
*Brevican* rev		cggtaccatggattgtgtttc		
*Collagen IV* for	*COL4A1*	gggagaaaagggtgaagca	NM_001845.5	72
*Collagen IV* rev		ccaaaggtcctgtgcctataa		
*Fibronectin* for	*FN1*	cttccacaggaggcctacac	NM_212482.1	114
			NM_002026.2	
			NM_212478.1	
*Fibronectin* rev		cgcaaaatatgctggaacttt	NM_212476.1	
			NM_212474.1	
*GAPDH* for	*GAPDH*	tccactggcgtcttcacc	NM_001289746.1	114
			NM_001289745.1	
*GAPDH* rev		ggcagagatgatgaccctttt	NM_001256799.2	
			NM_002046.5	
*α4-Integrin* for	*ITGA4*	gatgaaaatgagcctgaaacg	NM_000885.4	80
*α4-Integrin* rev		gccatactattgccagtgttga		
*α5-Integrin* for	*ITGA5*	cccattgaatttgacagcaa	NM_002205.2	92
*α5-Integrin* rev		tgcaaggacttgtactccaca		
*β1-Integrin* for	*ITGB1*	cgatgccatcatgcaagt	NM_002211	71
*β1-Integrin* rev		acaccagcagccgtgtaac	NM_133376	
*α1-Laminin* for	*LAMA1*	aggatgacctccattctgactt	NM_005559.3	60
*α1-Laminin* rev		ccttacatgggcactgacct		
*MMP-2* for	*MMP2*	ctacgaccgcgacaagaagt	NM_001302510.1	114
			NM_001302509.1	
			NM_001302508.1	
*MMP-2* rev		agttcccaccaacagtggac	NM_001127891.2	
			NM_004530.5	
*MMP-7* for	*MMP7*	gctgacatcatgattggcttt	NM_002423.3	113
*MMP-7* rev		tctcctccgagacctgtcc		
*MMP-9* for	*MMP9*	gtaccacggccaactacgac	NM_004994.2	74
*MMP-9* rev		gccgtcctgggtgtagagt		
*Neurocan* for	*NCAN*	aggggttcggagctatgg	NM_004386.2	95
*Neurocan* rev		ggcccacgtagaagacctc		
*S18* for	*RPS18*	cttccacaggaggcctacac	NM_022551.2	82
*S18* rev		cgcaaaatatgctggaacttt		
*TIMP-1* for	*TIMP1*	agtggcactcattgcttgtg	NM_003254.2	104
*TIMP-1* rev		ggactggaagcccttttca		
*TIMP-2* for	*TIMP2*	gaagagcctgaaccacaggt	NM_003255.4	85
*TIMP-2* rev		cggggaggagatgtagcac		
*Tenascin-C* for	*TNC*	gtctccagcctgccacag	NM_002160.3	82
*Tenascin-C* rev		cgcctttccagccaggttc		
*Tenascin-R* for	*TNR*	agtgacctctgagcccattg	NM_003285.2	61
*Tenascin-R* rev		gatgtcttttgggggatcaa		
*Versican* for	*VCAN*	tcccaggaaacttacgatg	NM_004385.4	70
*Versican* rev		ggggacagtgaggtgggaa		

**Table 2:** List of primer pairs used for semi-quantitative RT-PCR analyses of *RPTPβ/ζ* isoforms in WERI-RB1 and WERI-ETOR cells. For quantification of *RPTPβ/ζ* (*PTPRZ1*) mRNA levels, *ACTB* served as a housekeeping gene. For each primer, the primer sequence and the predicted amplicon size is indicated. Abbreviations: bp—base pairs, for—forward, rev—reverse.

Primer Name	Gene	Primer Sequence	Accession Number	Amplicon Size (bp)
*β-Actin* for	*ACTB*	tgacggggtcacccacactgtgcccatcta	NM_001101.3	661
*β-Actin* rev		ctagaagcatttgcggtggacgatggaggg		
CP *RPTPβ/ζ* for	*PTPRZ1*	agtgtgcaagtgcttgcctat	XM_005250519.1	555
CP *RPTPβ/ζ* rev		tgcagaatagtcactctgctg	XM_017012477.1	
EC *RPTPβ/ζ* for		tgcctacttcccaactgag	NM_002851.2	509
EC *RPTPβ/ζ* rev		cagcatgagtagtggac		
TEC *RPTPβ/ζ* for		tgcctacttcccaactgag	NM_001206839.1	369
TEC *RPTPβ/ζ* rev		attgctccgacatcatctg		2949
